# Biofilm Dynamics and Antimicrobial Resistance in Rabbit Odontogenic Infections: A One Health Perspective

**DOI:** 10.3390/pathogens15090963

**Published:** 2026-09-09

**Authors:** Ramona Ioana Stîngă, George Cosmin Nadăş

**Affiliations:** Department of Microbiology, Immunology and Epidemiology, Faculty of Veterinary Medicine, University of Agricultural Sciences and Veterinary Medicine, 400372 Cluj-Napoca, Romania; ioana.stinga@usamvcluj.ro

**Keywords:** rabbit odontogenic abscesses, biofilms, dental disease, antimicrobial resistance, oral microbiome, One Health

## Abstract

Rabbit odontogenic abscesses are among the most challenging chronic infections encountered in exotic animal medicine because of their polymicrobial etiology, biofilm-associated persistence, and poor response to conventional antimicrobial therapy. Biofilm formation plays a central role in disease pathogenesis by promoting bacterial adhesion, extracellular polymeric substance (EPS) production, quorum sensing (bacterial cell-to-cell communication), metabolic heterogeneity, and the persister-cell formation (transiently antibiotic-tolerant bacterial subpopulations), collectively reducing antimicrobial susceptibility and contributing to treatment failure and recurrence. In addition to biofilm-mediated tolerance, antimicrobial resistance (AMR) further complicates disease management through mechanisms including horizontal gene transfer, efflux pump activation, enzymatic antibiotic degradation, reduced membrane permeability, and target modification. This review summarizes current knowledge on the microbiology, biofilm dynamics, and resistance mechanisms associated with rabbit odontogenic infections while examining recent advances in molecular diagnostics, including culture-independent sequencing technologies, metagenomics, and advanced imaging approaches. Current and emerging anti-biofilm strategies, such as local antimicrobial delivery systems, enzymatic biofilm disruption, quorum-sensing inhibitors, bacteriophage therapy, antimicrobial peptides, photodynamic therapy, and nanotechnology-based approaches, are critically discussed in the context of their potential application in rabbits. Comparative evidence from human endodontic infections and other veterinary biofilm-associated diseases highlights the translational relevance of rabbit odontogenic abscesses as a naturally occurring model for chronic polymicrobial infections. Finally, key research gaps are identified, emphasizing the need for standardized experimental models, integrated multi-omics analyses, combining genomic, transcriptomic, proteomic, and metabolomic data, longitudinal clinical investigations, and evidence-based antimicrobial stewardship. By integrating microbiology, biofilm biology, antimicrobial resistance, and One Health concepts, this review provides a comprehensive framework to support future research and improve the diagnosis, treatment, and prevention of rabbit odontogenic infections.

## 1. Introduction

Odontogenic abscesses in rabbits (*Oryctolagus cuniculus*) are among the most challenging chronic infections encountered in exotic animal practice because of their complex pathogenesis, polymicrobial nature, and poor response to conventional antimicrobial therapy. Dental disease is one of the most common disorders affecting pet rabbits, with acquired conditions such as malocclusion, apical tooth elongation, and other dental abnormalities predisposing to odontogenic infection [1,2]. Once infection extends to the tooth apex and surrounding bone and soft tissues, abscesses frequently become polymicrobial, are commonly associated with osteomyelitis and bone lysis, and develop dense caseous exudate that severely limits drainage and antimicrobial penetration [3,4]. Consequently, these infections are associated with considerable morbidity, often requiring repeated surgical intervention and prolonged antimicrobial therapy, while recurrence remains common, ultimately compromising rabbit welfare and quality of life [5].

Treatment of rabbit odontogenic abscesses remains particularly challenging because successful management usually requires a multimodal approach rather than antimicrobial therapy alone. Management typically involves advanced imaging such as computed tomography (CT) and cone-beam computed tomography (CBCT) for accurate lesion assessment, extraction of affected teeth, surgical debridement, prolonged antimicrobial therapy, and close postoperative monitoring with sustained owner compliance. Clinical evidence further illustrates these challenges: in a retrospective study of 72 client-owned rabbits with odontogenic abscesses, systemic antibiotic therapy alone was associated with clinical resolution in approximately 25% of cases, whereas combining surgery, local wound packing, and antimicrobial treatment was associated with improved outcomes [3]. However, these findings should not be generalized across all clinical presentations, as outcomes may be influenced by disease severity and treatment-related factors, and complete resolution of infection was not systematically confirmed using advanced imaging. As pet rabbits become increasingly popular companion animals, the growing incidence of complex odontogenic disease places a considerable clinical, economic, and welfare burden on both owners and veterinary practitioners [6]. The limitations of current treatment strategies reflect both the biofilm-associated nature of these infections and the technical challenges of achieving adequate surgical debridement and postoperative local management, emphasizing the need for improved diagnostic approaches, targeted anti-biofilm therapies, and responsible antimicrobial stewardship.

The limited efficacy of conventional treatment strategies may partly reflect the biofilm-mediated nature of rabbit odontogenic abscesses. Rather than existing exclusively as free-floating (planktonic) bacterial cells, bacteria within these lesions may occur in structured biofilm communities embedded in a self-produced EPS matrix. This highly organized architecture is known in biofilm-associated infections to provide mechanical protection, restricts antimicrobial penetration, facilitate immune evasion, and enable bacterial persistence despite prolonged antimicrobial exposure [7]. Consequently, rabbit odontogenic abscesses share many pathophysiological features with chronic biofilm-associated dental and orthopedic infections described in other animal species and humans. Several bacterial species isolated from rabbit abscesses, including *Pseudomonas aeruginosa*, *Stenotrophomonas maltophilia*, *Pasteurella multocida*, and *Staphylococcus aureus*, have been associated with biofilm-forming capacity and antimicrobial resistance [4,5,8]. However, direct rabbit-specific evidence linking biofilm formation or efflux pump activity to treatment failure and recurrence remains limited, and these mechanisms should therefore not be considered established determinants of clinical recurrence in rabbit odontogenic abscesses.

The biofilm phenotype profoundly alters microbial physiology, promoting bacterial persistence and antimicrobial tolerance. Within the dense, poorly vascularized, caseous environment characteristic of rabbit abscesses, oxygen gradients and nutrient limitation are expected to create conditions that favor the emergence of metabolically dormant “persister” cells, which exhibit transient tolerance to antibiotic concentrations far exceeding those required to eliminate their planktonic counterparts [9]. These considerations may also extend to the postoperative period, during which adequate exposure of healing tissues and restoration of local vascular support may improve antimicrobial delivery and contribute to infection control. Together with quorum-sensing-mediated communication, these phenotypic adaptations may promote coordinated virulence, immune evasion, and recolonization following apparently successful therapy. Although these mechanisms have been extensively characterized in human endodontic and osteomyelitic biofilms, they provide a biologically plausible framework for understanding the chronicity and treatment failure associated with rabbit odontogenic abscesses, highlighting the translational relevance of this disease model [10,11].

Beyond promoting antimicrobial tolerance, biofilm formation may also contribute to the emergence and dissemination of AMR in rabbit odontogenic infections. The EPS matrix limits antimicrobial penetration, while the close spatial organization of multispecies biofilms may facilitate horizontal gene transfer and, together with sustained selective antibiotic pressure, may favor the selection and persistence of multidrug-resistant bacterial populations [12]. These mechanisms have important One Health implications because several of the bacterial genera commonly associated with rabbit odontogenic abscesses, including *Pseudomonas*, *Burkholderia*, and *Staphylococcus*, circulate across animal, human, and environmental reservoirs, creating opportunities for the dissemination of resistance determinants. Consequently, a better understanding of the interactions between biofilm biology and AMR is essential for the development of more effective local therapeutic strategies and for strengthening antimicrobial stewardship in exotic animal medicine [13,14].

Despite growing recognition of the importance of biofilm formation and antimicrobial resistance in rabbit odontogenic infections, current knowledge remains fragmented across veterinary dentistry, microbiology, and One Health research. Most published studies focus on individual aspects of disease pathogenesis, bacterial identification, or therapeutic management. At the same time, an integrated perspective that links biofilm biology, antimicrobial resistance mechanisms, molecular diagnostics, and emerging treatment strategies remains lacking. This review aims to provide a comprehensive synthesis of current evidence on the microbiology and biofilm dynamics of rabbit odontogenic abscesses, examine the mechanisms underlying antimicrobial resistance and persistence, discuss advances in molecular diagnostic approaches and novel therapeutic options, and highlight the broader One Health implications of these chronic infections.

**Figure 1:**
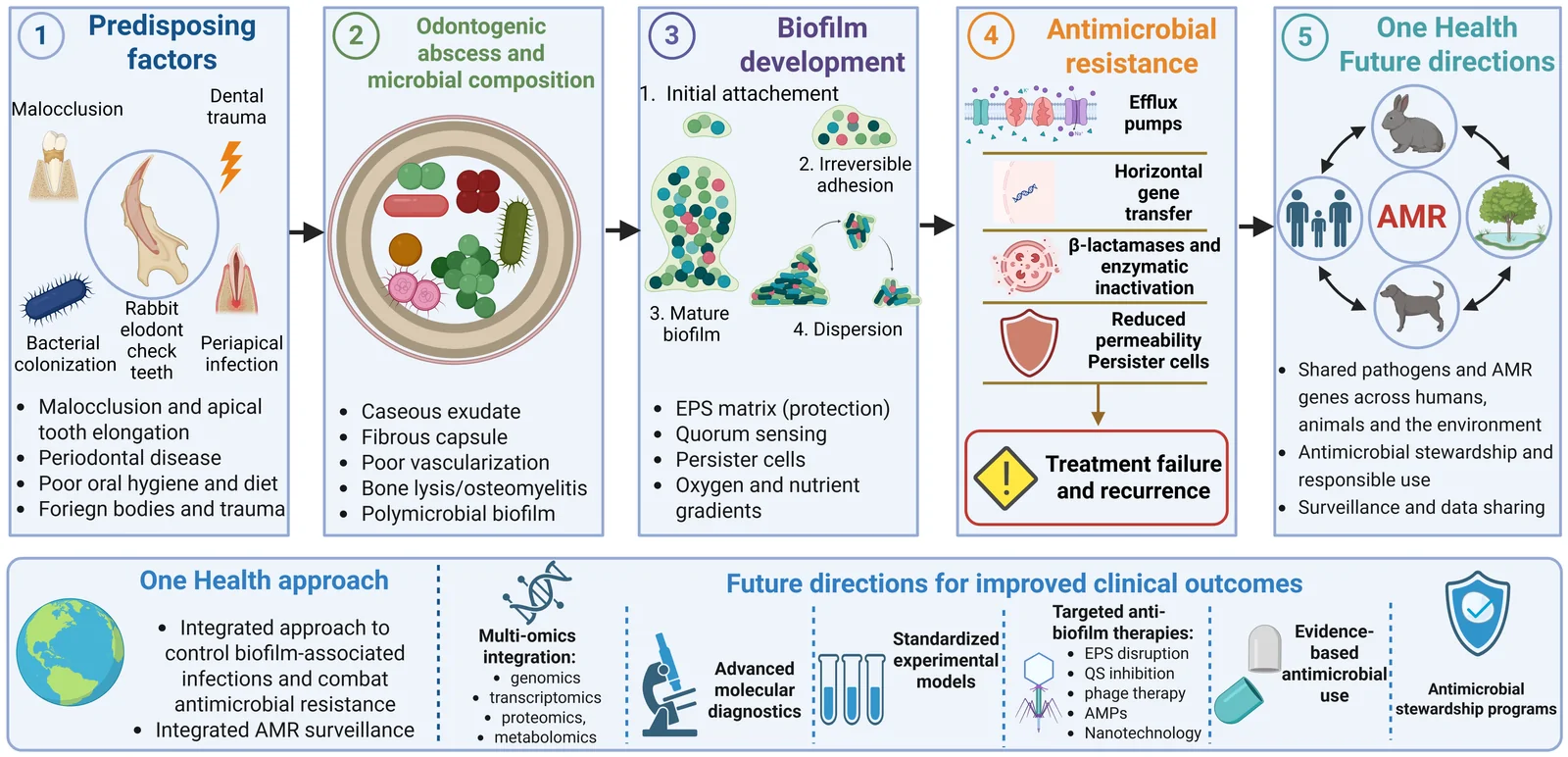
Conceptual overview of biofilm dynamics and antimicrobial resistance in rabbit odontogenic infections within a One Health framework. Predisposing dental abnormalities facilitate the development of polymicrobial odontogenic abscesses, where biofilm maturation promotes persistence through extracellular polymeric substance (EPS) production, quorum sensing, oxygen and nutrient gradients, and persister-cell formation. Biofilm-associated antimicrobial resistance contributes to treatment failure and recurrence, highlighting the need for integrated One Health strategies based on molecular diagnostics, multi-omics technologies, innovative anti-biofilm therapies, and antimicrobial stewardship to improve clinical outcomes [Created in BioRender. Nadas, N. (2026) https://BioRender.com/6dont2u].

The major concepts discussed throughout this review, including the progression from predisposing factors to biofilm formation, antimicrobial resistance, and the One Health implications of rabbit odontogenic infections, are summarized in Figure 1.

## 2. Literature Search Strategy

This review was conducted as a narrative literature review. A comprehensive search of the scientific literature was performed using PubMed/MEDLINE, Scopus, Web of Science, and Google Scholar to identify relevant studies published from database inception through April 2026, with no lower publication-date restriction. Search terms included combinations of “rabbit”, “odontogenic abscess”, “dental abscess”, “oral biofilm”, “biofilm”, “antimicrobial resistance”, “odontogenic infection”, “oral microbiome”, “One Health”, “photodynamic therapy”, “antimicrobial stewardship”, and related keywords. Boolean operators (AND, OR) were used to refine the search strategy.

Original research articles, systematic reviews, meta-analyses, clinical guidelines, and authoritative review articles published in English were considered. Priority was given to studies specifically addressing rabbit odontogenic infections; however, because of the limited species-specific literature, evidence from human endodontic infections and relevant veterinary biofilm-associated diseases was also included to provide a comprehensive and comparative One Health perspective. Articles were selected based on their scientific relevance, methodological quality, and contribution to the topics discussed in this review. When multiple publications addressed the same topic, priority was given to rabbit-specific studies, original research, studies providing direct experimental or clinical evidence, and more recent or methodologically comprehensive publications. Where direct evidence in rabbits was lacking, comparative evidence from human dentistry and other animal species was incorporated to highlight conserved mechanisms of biofilm development, antimicrobial resistance, and chronic odontogenic infection.

## 3. Biofilm Biology in Rabbit Odontogenic Infections

The unique structural organization of rabbit odontogenic abscesses may provide a favorable microenvironment for biofilm establishment and persistence. Unlike the liquefactive abscesses commonly observed in many mammalian species, most rabbit abscesses are characterized by dense caseous exudate enclosed within a thick fibrous capsule and surrounded by poorly vascularized tissue [15,16]. This distinctive architecture restricts drainage, limits systemic antimicrobial penetration, and may promote the development of localized physicochemical gradients that favor chronic bacterial survival.

Biofilms are structured microbial communities embedded within a self-produced EPS matrix that adheres to biological or abiotic surfaces [16]. Rather than representing simple bacterial aggregates, biofilms function as highly organized multicellular systems in which microorganisms coordinate gene expression, metabolic activity, and interactions with the surrounding microenvironment. In rabbit odontogenic abscesses, biofilm formation is considered an important contributor to disease persistence. The dense caseous exudate, fibrous capsule, and limited vascularization may create a protected niche that favors bacterial attachment, EPS production, and the establishment of stable polymicrobial communities. Once established, these biofilms may contribute to chronic infection by restricting antimicrobial penetration, reducing bacterial metabolic activity, promoting immune evasion, and serving as reservoirs for persistent and recurrent infection [16,17].

Biofilm development is a dynamic, multistep process comprising initial attachment, irreversible adhesion, microcolony formation, maturation, and eventual dispersion [16]. Initial bacterial attachment is mediated by physicochemical interactions and surface-associated structures, including pili and flagella, while subsequent production of EPS stabilizes bacterial adhesion and promotes the formation of highly organized three-dimensional communities [18,19]. In rabbit odontogenic abscesses, the exposed dental tissues, necrotic bone, and caseous exudate may provide favorable substrates for bacterial colonization, potentially facilitating the transition from planktonic growth to mature biofilm formation.

Following initial attachment, bacterial adhesion becomes irreversible through the production of EPS, which anchors cells to the surface and provides the structural scaffold for biofilm development [19,20]. As bacterial proliferation continues, adherent cells organize into microcolonies embedded within the EPS matrix. In established biofilm models, at this stage, cell-to-cell communication through quorum sensing coordinates gene expression, regulates EPS production, and promotes the transition to a mature, highly structured biofilm. In rabbit odontogenic abscesses, the poorly vascularized, caseous environment is likely to facilitate this transition by providing a stable niche that supports long-term microbial persistence [20].

Mature biofilms comprise metabolically diverse microbial subpopulations that interact through cooperative networks rather than functioning as isolated bacterial cells. These interactions facilitate nutrient exchange, metabolic complementation, and coordinated adaptation to environmental stress, thereby enhancing the stability and resilience of the microbial community [21]. In rabbit odontogenic abscesses, where polymicrobial infections are common, such metabolic cooperation may contribute to bacterial persistence, the coexistence of aerobic and anaerobic species, and increased tolerance to both host immune responses and antimicrobial therapy.

During the maturation stage, biofilms develop into highly organized three-dimensional structures in which bacterial cells are embedded within an extensive EPS matrix. At this stage, quorum sensing plays a central regulatory role by coordinating gene expression involved in biofilm maintenance, EPS production, stress adaptation, and the expression of virulence-associated traits [17]. As the biofilm thickens, chemical gradients of oxygen, nutrients, and metabolic waste create physiologically distinct microenvironments that support the coexistence of metabolically active and dormant bacterial subpopulations [22]. In rabbit odontogenic abscesses, the dense caseous matrix and limited vascularization may further accentuate these gradients, potentially contributing to biofilm resilience, antimicrobial tolerance, and chronic infection [15].

As maturation progresses, quorum sensing and other intercellular signaling mechanisms coordinate the spatial organization of bacterial populations, resulting in a highly structured three-dimensional biofilm with distinct physiological niches [23]. The final stage of the biofilm life cycle is dispersion, an active process in which individual bacterial cells or multicellular aggregates detach from the mature biofilm and colonize new sites [24]. Dispersion contributes to the persistence and recurrence of chronic infections by enabling microorganisms to establish new biofilm foci within the infected tissue. In rabbit odontogenic abscesses, this process may facilitate recolonization of residual infected tissue following surgical debridement and could therefore contribute to treatment failure and recurrence.

Rabbit heterophils differ functionally from the neutrophils of many other mammalian species and represent a key determinant of the distinctive inflammatory response observed in rabbit abscesses. Histologically, they are often referred to as pseudoeosinophils because of their numerous eosinophilic cytoplasmic granules [25]. These granules comprise primary (azurophilic) and secondary (specific) granules but contain lower levels of neutral proteases and myeloperoxidase (MPO) than those of human or canine neutrophils [25,26]. Consequently, rabbit heterophils generate a less efficient MPO-dependent oxidative burst and reduced hypochlorous acid production, limiting tissue liquefaction and favoring the formation of the dense caseous exudate that characterizes rabbit odontogenic abscesses [2,27].

Microscopic analyses have substantially advanced the understanding of biofilm architecture in odontogenic infections. Although direct imaging studies in rabbits remain limited, available scanning electron microscopy (SEM) and histopathological investigations have identified dense bacterial aggregates embedded within a heterogeneous EPS matrix closely associated with necrotic debris and mineralized dental tissues. Owing to the scarcity of rabbit-specific imaging data, much of the current understanding of biofilm ultrastructure is supported by studies in human and other small-animal odontogenic infections. These investigations, using SEM and confocal laser scanning microscopy (CLSM), consistently demonstrate highly organized three-dimensional biofilms characterized by microcolonies, water channels, and spatially heterogeneous bacterial populations [20,22].

CLSM studies further demonstrate the presence of physiologically distinct bacterial subpopulations within mature biofilms, including dormant persister cells located in deeper layers, which contribute to antimicrobial tolerance and the persistence of chronic infections [28]. Similar microscopy-based investigations in canine and feline abscesses have confirmed polymicrobial biofilms closely associated with tissue interfaces and implanted materials, reinforcing the concept that biofilm formation is a major driver of chronic abscess persistence. Extrapolating these findings to rabbit odontogenic abscesses, the combination of a dense caseous matrix, limited vascularization, and a sustained inflammatory microenvironment is likely to promote the development of structurally complex and highly resilient biofilms [25]. However, direct visualization of biofilm architecture in rabbits remains scarce, highlighting the need for further studies employing SEM, CLSM, and complementary imaging techniques to better characterize these infections and support the development of targeted anti-biofilm therapies.

## 4. Microbial Ecology of Rabbit Odontogenic Biofilms

Rabbit odontogenic abscesses are polymicrobial infections in which disease progression is driven by complex microbial communities rather than by a single pathogen. Culture-based studies have consistently identified a diverse consortium of aerobic and anaerobic bacteria, including *Pasteurella multocida*, *Staphylococcus* spp., *Pseudomonas* spp., *Bacteroides* spp., *Fusobacterium* spp., *Escherichia coli*, and *Proteus* spp., reflecting the ecological complexity of these lesions [15]. Early investigations by Tyrrell et al. further demonstrated the important contribution of anaerobic bacteria to rabbit odontogenic infections and the polymicrobial nature of these lesions, identifying *Fusobacterium nucleatum*, *Actinomyces* spp., and members of the *Streptococcus milleri* group among key components of the microbial community [29]. Rather than acting independently, these microorganisms are likely to cooperate metabolically and structurally within the biofilm, potentially contributing to bacterial persistence, immune evasion, and increased tolerance to antimicrobial therapy.

The biofilm matrix provides an ideal environment for horizontal gene transfer (HGT), facilitating the dissemination of antimicrobial resistance determinants within polymicrobial bacterial communities. The dense EPS matrix maintains prolonged cell-to-cell contact, increasing opportunities for plasmid conjugation and the exchange of mobile genetic elements compared with planktonic bacteria [30]. In addition, the heterogeneous biofilm microenvironment, characterized by nutrient limitation, oxygen gradients, and exposure to sub-inhibitory antimicrobial concentrations, promotes bacterial stress responses that may further enhance genetic adaptation and the acquisition of resistance traits. In rabbit odontogenic abscesses, where dense polymicrobial biofilms persist within poorly vascularized caseous lesions, these conditions may facilitate horizontal gene transfer and could thereby contribute to the emergence and persistence of multidrug-resistant bacterial populations [22,28].

In addition to horizontal gene transfer, biofilm-associated bacteria employ multiple complementary mechanisms, including efflux pump activation, enzymatic antibiotic degradation, target modification, and persister-cell formation, that collectively reduce antimicrobial susceptibility and promote chronic infection [31]. The principal bacterial species associated with rabbit odontogenic abscesses, together with their microbiological characteristics, biofilm-forming capacity, and major antimicrobial resistance features, are summarized in Table 1.

## 5. Mechanisms of Antimicrobial Resistance Within Rabbit Dental Biofilms

Antimicrobial resistance in rabbit odontogenic biofilms may reflect the combined action of intrinsic and acquired bacterial mechanisms operating within the protective biofilm environment. In biofilm-associated infections, biofilm architecture promotes bacterial persistence and horizontal gene transfer; individual microorganisms further reduce antimicrobial susceptibility through complementary resistance strategies, including efflux pump activation, reduced membrane permeability, enzymatic antibiotic degradation, target modification, quorum-sensing-mediated adaptation, and persister-cell formation. Together, these mechanisms may limit antimicrobial efficacy and contribute to the chronicity and recurrence of rabbit odontogenic abscesses [31,32,33]. The principal mechanisms relevant to biofilm-associated infections are discussed below.

### 5.1. Efflux Pumps

Efflux pump activation is one of the principal mechanisms by which biofilm-associated bacteria reduce intracellular antibiotic accumulation and survive antimicrobial exposure. These membrane-associated transport systems actively export a wide range of antimicrobial agents, lowering intracellular drug concentrations below bactericidal levels and thereby reducing bacterial susceptibility [33,34]. Beyond their direct role in antibiotic extrusion, efflux pumps also influence fundamental bacterial physiology, stress adaptation, and biofilm development, contributing to the persistence of chronic infections [34]. Sustained efflux pump activity during antimicrobial exposure may promote the emergence of intermediate resistance phenotypes, allowing bacterial populations to survive long enough to acquire additional adaptive mutations or resistance determinants [35]. In rabbit odontogenic abscesses, where prolonged antimicrobial therapy is frequently combined with dense polymicrobial biofilms, efflux pump activation may contribute to treatment failure and bacterial persistence, particularly among *Pseudomonas aeruginosa*, *Staphylococcus aureus*, and other opportunistic pathogens commonly isolated from these lesions [4,7].

### 5.2. β-Lactamases

Enzymatic degradation of antimicrobial agents represents another major mechanism of resistance in biofilm-associated infections. Among these enzymes, β-lactamases are the most clinically important because they hydrolyze β-lactam antibiotics, including penicillins, cephalosporins, carbapenems, and monobactams, thereby preventing these drugs from reaching their target penicillin-binding proteins (PBPs) and inhibiting bacterial cell wall synthesis [36,37]. The extensive use of β-lactam antibiotics has driven the evolution and dissemination of β-lactamases through both horizontal gene transfer and spontaneous mutation, resulting in an expanding diversity of enzymes with enhanced substrate spectra and contributing substantially to the global spread of antimicrobial resistance [38]. The widespread production of β-lactamases among Gram-negative and Gram-positive pathogens further compromises the efficacy of β-lactam therapy and has accelerated the emergence of multidrug-resistant bacteria [37,38]. Within polymicrobial biofilms, β-lactamase-producing bacteria may also protect neighboring susceptible microorganisms by reducing local antibiotic concentrations, thereby enhancing community-level antimicrobial tolerance [11,31]. In rabbit odontogenic abscesses, where β-lactam antibiotics are frequently administered and polymicrobial biofilms predominate, enzymatic antibiotic degradation is therefore likely to contribute to treatment failure and infection persistence, although rabbit-specific evidence remains limited [4,7].

### 5.3. Quorum Sensing

Quorum sensing (QS) is a bacterial cell-to-cell communication system that coordinates gene expression in response to population density, enabling microbial communities to adapt collectively to changing environmental conditions [39]. Through the production and detection of small signaling molecules, QS regulates multiple biological processes associated with bacterial persistence, including biofilm maturation, virulence factor production, motility, stress adaptation, and antimicrobial resistance [39,40]. In polymicrobial biofilms, QS also coordinates interactions between different bacterial species, optimizing community organization and enhancing survival under antimicrobial pressure [41,42,43,44].

One of the most important contributions of QS to antimicrobial resistance is the regulation of biofilm development through the coordinated production of EPSs, which form the protective matrix surrounding bacterial communities [41,42,43,44]. This matrix restricts antibiotic penetration, reduces bacterial exposure to host immune defenses, and creates physiologically heterogeneous microenvironments that favor bacterial persistence and antimicrobial tolerance [11,31]. In rabbit odontogenic abscesses, where chronic polymicrobial biofilms may contribute to treatment failure, QS is therefore likely to play a central role in maintaining biofilm stability and promoting persistent infection, although direct evidence in rabbits remains limited [4,7].

### 5.4. Persister Cells

Persister cells are a small phenotypic subpopulation of bacteria that survive antimicrobial exposure without acquiring stable, heritable resistance. Unlike resistant mutants, persister cells remain genetically susceptible and regain normal antimicrobial susceptibility after exiting dormancy and resuming active growth [45]. Their clinical significance is particularly evident in chronic biofilm-associated infections, where metabolically inactive cells survive prolonged antimicrobial treatment within protected biofilm niches and subsequently repopulate the lesion once therapy is discontinued, contributing to recurrent infection [45].

Evidence from human odontogenic biofilms supports a central role for phenotypic tolerance in persistent infection. Mature endodontic biofilms contain physiologically heterogeneous bacterial populations exposed to steep gradients of nutrients, oxygen, and pH, creating microenvironments that favor slow-growing or dormant cells with reduced susceptibility to antimicrobial agents and disinfectants [46]. These protected bacterial subpopulations are increasingly recognized as major contributors to treatment failure and recurrence of infection in chronic odontogenic infections [46]. Comparable conditions may occur in rabbit odontogenic abscesses, where dense caseous exudate, poor vascularization, and mature polymicrobial biofilms are expected to promote persister-cell formation and long-term bacterial survival, although direct experimental evidence in rabbits remains scarce [4,7,11].

At the molecular level, persister-cell formation is regulated by multiple stress-response pathways that have been extensively characterized in bacterial species associated with chronic biofilm infections but remain largely unexplored in rabbit odontogenic disease. These include activation of the stringent response mediated by guanosine tetraphosphate and pentaphosphate ((p)ppGpp) under nutrient limitation, toxin–antitoxin systems that transiently suppress bacterial growth and protein synthesis, and global stress-response networks associated with metabolic downregulation and biofilm adaptation [47]. Collectively, these regulatory pathways shift a subpopulation of bacterial cells into a metabolically inactive or slow-growing state in which the cellular targets of antimicrobial agents become less susceptible to antibiotic-mediated killing, thereby promoting persistence during antimicrobial therapy [45,47]. Although these molecular mechanisms have not yet been specifically investigated in rabbit odontogenic abscesses, the chronic, nutrient-limited, and biofilm-rich microenvironment of these lesions is expected to provide conditions favorable for persister-cell formation and long-term bacterial survival [11,31].

Experimental rodent models further support the biological importance of persister cells in chronic biofilm-associated infections. In murine *Staphylococcus aureus* biofilm models, persister-enriched bacterial populations exhibit prolonged survival within the host and reduced susceptibility to immune-mediated clearance, demonstrating that phenotypic tolerance contributes directly to chronic infection persistence in vivo [48]. Similar findings have been reported in rodent osteomyelitis models, where *Staphylococcus aureus* biofilm formation and persistence within infected tissues contribute to the establishment of chronic infection [48]. Although comparable experimental studies are currently lacking in rabbits, these observations provide strong biological support for the hypothesis that persister cells contribute to the chronicity and therapeutic recalcitrance of rabbit odontogenic abscesses, where mature biofilms, poor vascularization, and prolonged antimicrobial exposure create comparable selective pressures [4,7,11,31].

Although persister cells have not yet been directly demonstrated in rabbit odontogenic abscesses, the pathological characteristics of these lesions strongly support their biological plausibility. Rabbit abscesses are characterized by a dense caseous core, poor vascularization, restricted diffusion of oxygen and nutrients, and chronic inflammatory remodeling, creating a microenvironment expected to generate the metabolic heterogeneity that favors persister-cell formation in human endodontic biofilms and experimental rodent models of chronic biofilm-associated infection [45,46]. Consequently, the involvement of persister cells in rabbit odontogenic abscesses should currently be regarded as a biologically plausible hypothesis supported by comparative biofilm research rather than as a mechanism that has been experimentally demonstrated in this species [45,46].

## 6. Diagnostic and Molecular Approaches for Rabbit Odontogenic Biofilms

### 6.1. Conventional Culture-Based Diagnostics

Accurate characterization of the microbial communities associated with rabbit odontogenic abscesses remains a major diagnostic challenge because these lesions are polymicrobial, biofilm-associated, and frequently contain fastidious or anaerobic microorganisms. Consequently, conventional culture-based methods, although routinely employed in veterinary practice, often fail to capture the full diversity of bacterial populations present within these complex infections. In biofilm-associated infections, bacteria embedded within mature biofilms or existing in viable but non-culturable (VBNC) states may escape detection, leading to an underestimation of both microbial diversity and clinically relevant pathogens [20,49]. These diagnostic limitations may hinder targeted antimicrobial selection, obscure polymicrobial interactions, and reduce our understanding of the microbial ecology underlying chronic rabbit odontogenic abscesses.

### 6.2. 16S rRNA Gene Sequencing

To overcome the inherent limitations of culture-based diagnostics, culture-independent molecular approaches have become essential tools for characterizing the microbial communities associated with biofilm-related infections. Techniques including 16S rRNA gene sequencing, quantitative PCR (qPCR), shotgun metagenomics, and advanced imaging methods enable comprehensive characterization of microbial composition, functional potential, interspecies interactions, and antimicrobial resistance determinants that are often overlooked by conventional microbiological methods [50,51]. Their application in human dental and orthopedic biofilm research has substantially expanded our understanding of polymicrobial community structure, biofilm ecology, and the distribution of resistance genes, demonstrating the limitations of relying exclusively on culture-based diagnostics.

Despite these advances, the application of molecular diagnostic approaches to rabbit odontogenic abscesses remains limited. Given the polymicrobial and biofilm-associated nature of these infections, integrating molecular techniques into routine investigations could improve pathogen detection, support more targeted antimicrobial selection, and provide valuable insights into the ecology and dissemination of antimicrobial resistance. Moreover, identifying shared microbial communities and resistance determinants across companion animals, humans, and environmental reservoirs would strengthen the One Health perspective and facilitate surveillance of emerging resistance mechanisms [52,53].

16S rRNA gene sequencing has become one of the most widely used culture-independent approaches for characterizing the microbial composition of biofilm-associated infections. By targeting conserved and hypervariable regions of the bacterial 16S rRNA gene, this technique enables comprehensive profiling of complex polymicrobial communities, including fastidious and previously unrecognized bacteria that are frequently overlooked by conventional culture methods [49,50]. This capability is particularly relevant for odontogenic abscesses, where structured biofilms harbor diverse aerobic and anaerobic microorganisms whose interactions contribute to disease persistence. In human endodontic and periodontal infections, 16S rRNA sequencing has substantially expanded our understanding of microbial community structure, revealing highly diverse polymicrobial consortia that include *Fusobacterium*, *Prevotella*, and numerous uncultured anaerobic taxa often underrepresented or undetected by routine microbiological techniques [20]. These findings have highlighted the complexity of oral biofilms and demonstrated that culture-based diagnostics frequently underestimate both microbial diversity and the ecological interactions that influence biofilm development and chronic infection.

Although the application of 16S rRNA sequencing to rabbit odontogenic abscesses remains limited, this approach offers considerable potential to improve the characterization of their microbial ecology. The polymicrobial and biofilm-associated nature of these infections, together with the frequent involvement of fastidious and anaerobic bacteria, suggests that conventional culture-based diagnostics underestimate microbial diversity and incompletely characterize the ecological interactions occurring within the abscess microenvironment [2,4]. By providing a more comprehensive profile of bacterial communities, 16S rRNA sequencing may facilitate the identification of previously undetected taxa, improve our understanding of microbial community structure, and clarify the ecological relationships that contribute to biofilm persistence and disease progression.

Beyond improving microbial identification, 16S rRNA sequencing facilitates comparative analyses of biofilm-associated microbial communities across host species. As molecular data become available for rabbit odontogenic abscesses, comparisons with human endodontic and periodontal biofilms may help identify conserved microbial taxa, ecological patterns, and pathogenic mechanisms, thereby strengthening the translational relevance of rabbit odontogenic infections as a model of chronic biofilm-associated disease. Despite its advantages, 16S rRNA sequencing has important limitations. The method generally provides limited taxonomic resolution at the species or strain level and does not directly characterize the functional capacity of microbial communities or detect antimicrobial resistance genes. Consequently, 16S-based profiling is best complemented by shotgun metagenomic sequencing, which enables simultaneous characterization of microbial composition, functional pathways, and the resistome [51].

### 6.3. qPCR and Shotgun Metagenomics

While 16S rRNA sequencing provides valuable information on microbial community composition, it offers limited insight into the functional characteristics of these communities, particularly their antimicrobial resistance profiles. qPCR and shotgun metagenomic sequencing therefore represent complementary molecular approaches for detecting and characterizing antimicrobial resistance determinants within biofilm-associated infections. qPCR enables the rapid, sensitive, and quantitative detection of predefined resistance genes, making it particularly useful for identifying clinically relevant determinants such as *bla_TEM_*, *tetA*, and *sul1*, which are associated with resistance to β-lactams, tetracyclines, and sulfonamides, respectively. Because of its speed and high analytical sensitivity, qPCR is well suited for targeted screening of known resistance markers and may support antimicrobial selection and stewardship in clinical practice [52,53].

Unlike targeted molecular assays, shotgun metagenomic sequencing provides an untargeted characterization of the entire genetic content of microbial communities, enabling simultaneous analysis of taxonomic composition, functional potential, and the resistome. In addition to identifying known and novel antimicrobial resistance genes, this approach detects mobile genetic elements, including plasmids, transposons, and integrons, that facilitate horizontal gene transfer within polymicrobial biofilms [50,51]. In human dental and chronic wound biofilms, shotgun metagenomics has revealed complex reservoirs of antimicrobial resistance genes embedded within polymicrobial communities, frequently associated with virulence determinants and biofilm-related pathways. These findings have substantially improved our understanding of the ecological interactions that promote microbial persistence, adaptation, and the dissemination of antimicrobial resistance within chronic biofilm-associated infections [11,50].

Although the application of qPCR and shotgun metagenomics to rabbit odontogenic abscesses remains limited, these approaches have considerable potential to advance our understanding of the molecular epidemiology of these infections. Increasing reports of multidrug-resistant bacteria in pet rabbits highlight the need for diagnostic methods capable of identifying not only bacterial species but also the antimicrobial resistance determinants they harbor [2,4]. In the context of polymicrobial, biofilm-associated infections, molecular profiling could characterize the distribution of resistance genes within complex microbial communities and identify mobile genetic elements, including plasmids and integrons, that facilitate horizontal gene transfer. Such information would improve our understanding of the mechanisms driving the emergence and dissemination of antimicrobial resistance in rabbit odontogenic biofilms while supporting comparative studies across animal, human, and environmental reservoirs within a One Health framework [52].

Together, qPCR and shotgun metagenomics extend molecular diagnostics beyond taxonomic identification, providing functional information on antimicrobial resistance and microbial adaptation that is essential for understanding the pathogenesis of chronic biofilm-associated infections and for developing more targeted diagnostic and therapeutic strategies.

### 6.4. Biofilm Imaging Techniques

While sequencing-based approaches provide detailed information on microbial composition and genetic potential, they do not preserve the spatial organization of microbial communities within intact biofilms. Because biofilm architecture critically influences microbial interactions, nutrient gradients, antimicrobial penetration, and bacterial persistence, imaging techniques have become indispensable complements to molecular diagnostics. Among these, CLSM enables high-resolution, three-dimensional visualization of intact biofilms, revealing structural features such as microcolonies, water channels, and the distribution of the EPS matrix. In combination with fluorescent viability staining, CLSM also distinguishes viable from non-viable bacterial populations and provides insight into the physiological heterogeneity that underlies biofilm resilience and antimicrobial tolerance [11,28].

Complementing CLSM, fluorescence in situ hybridization (FISH) uses fluorescently labeled oligonucleotide probes targeting ribosomal RNA sequences to identify and localize specific bacterial taxa directly within intact biofilms. By preserving the spatial relationships between microorganisms, FISH enables the investigation of microbial ecology in situ, revealing species-specific distribution patterns, interspecies interactions, and niche specialization that cannot be inferred from sequencing data alone. In human dental plaque and endodontic biofilms, FISH has demonstrated highly organized polymicrobial communities in which aerobic and anaerobic bacteria occupy distinct but functionally interconnected microenvironments, supporting metabolic cooperation, biofilm stability, and persistence [20]. When combined with CLSM, FISH links microbial identity to biofilm architecture, allowing simultaneous visualization of community composition and three-dimensional organization. This integrated approach provides unique insights into the spatial organization of polymicrobial biofilms and the ecological interactions that contribute to chronic infection, antimicrobial tolerance, and biofilm resilience [20,54].

The application of CLSM and FISH to rabbit odontogenic abscesses remains extremely limited, representing an important gap in our understanding of the spatial organization of these infections [2,4,7]. Given the characteristic caseous matrix, poor vascularization, and polymicrobial nature of rabbit abscesses, in situ imaging has the potential to reveal how bacterial communities are organized within the lesion, how they interact with host tissues, and how biofilm architecture contributes to chronic infection [2,15,20]. These techniques could identify protected microenvironments that favor bacterial persistence, including niches likely to harbor persister cells, while providing insight into the structural basis of antimicrobial tolerance and treatment failure [11,20,28]. Integrating CLSM and FISH with sequencing-based approaches, including 16S rRNA gene sequencing and shotgun metagenomics, would provide a multidimensional characterization of rabbit odontogenic biofilms by linking microbial identity, functional potential, and spatial organization. Such integrated analyses would improve our understanding of biofilm ecology and support the development of more targeted diagnostic and anti-biofilm therapeutic strategies within a One Health framework [54].

### 6.5. MALDI-TOF MS and Next-Generation Sequencing

Matrix-assisted laser desorption/ionization time-of-flight mass spectrometry (MALDI-TOF MS) has become an important diagnostic tool in clinical microbiology by enabling rapid and accurate species-level identification of bacterial isolates based on unique protein spectral fingerprints. Compared with conventional biochemical methods, MALDI-TOF substantially reduces turnaround time while maintaining high diagnostic accuracy, thereby facilitating earlier antimicrobial selection and improving laboratory workflow [55,56]. In veterinary medicine, including small-animal practice, MALDI-TOF has enhanced the identification of bacterial pathogens recovered from abscesses and wound infections, including *Staphylococcus*, *Pseudomonas*, and *Pasteurella* species commonly associated with rabbit odontogenic disease [4,7]. However, because MALDI-TOF is inherently culture-dependent, it cannot detect uncultivable organisms or fully characterize polymicrobial biofilm communities. Its diagnostic performance also relies on the completeness of reference spectral databases, which remain limited for many exotic animal pathogens, fastidious microorganisms, and strict anaerobes, potentially leading to misidentification or underrepresentation of clinically relevant taxa [55,56].

Complementing MALDI-TOF MS, next-generation sequencing (NGS) technologies provide culture-independent genomic analyses that overcome many of the limitations associated with conventional microbiology and proteomic identification. Whole-genome sequencing (WGS) and metagenomic sequencing enable high-resolution pathogen identification, strain-level differentiation, and comprehensive characterization of virulence factors, antimicrobial resistance genes, and mobile genetic elements, providing information that cannot be obtained through protein-based identification alone [50,51]. In both human and veterinary medicine, NGS has proven particularly valuable for the investigation of complex polymicrobial infections, where it facilitates the detection of unculturable microorganisms, characterizes the functional potential of microbial communities, and supports epidemiological surveillance of antimicrobial resistance.

In rabbit odontogenic abscesses, integrating MALDI-TOF MS with NGS could substantially improve diagnostic workflows by combining the rapid identification of cultivable pathogens with comprehensive genomic characterization of polymicrobial communities [4,7,55,56]. While MALDI-TOF provides timely species-level identification to support routine clinical decision-making, NGS complements this information by identifying unculturable microorganisms; characterizing antimicrobial resistance determinants, virulence-associated genes, and mobile genetic elements; and revealing the functional complexity of biofilm-associated infections [50,51]. Expanding reference databases to include lagomorph-associated microorganisms would further improve diagnostic accuracy for exotic animal pathogens, particularly fastidious and anaerobic bacteria that remain underrepresented in current databases [55,56]. Beyond improving clinical management, these complementary technologies could strengthen molecular surveillance of antimicrobial resistance and facilitate comparative studies across companion animals, humans, and environmental reservoirs, reinforcing the One Health perspective [52,53].

## 7. Comparative and One Health Perspective

Human endodontic infections are among the best-characterized models of polymicrobial biofilm-associated disease and therefore provide a valuable comparative framework for understanding rabbit odontogenic abscesses. Disease progression from reversible pulpitis to apical periodontitis is accompanied by dynamic ecological shifts within complex polymicrobial biofilms, driven by changes in nutrient availability, oxygen tension, and host immune pressure rather than by the proliferation of a single pathogen [20,57,58]. The mature endodontic microbiota is typically dominated by obligate anaerobes, including *Porphyromonas*, *Prevotella*, *Fusobacterium*, and *Treponema* spp., several of which have also been identified in rabbit odontogenic abscesses [29]. Although rabbit abscesses are distinguished by their thick fibrous capsule and caseous exudate, the substantial overlap in dominant anaerobic taxa suggests that similar ecological interactions, metabolic cooperation, and biofilm-driven persistence mechanisms may underlie chronic infection in both species [20,21,22,59]. These similarities support the use of human endodontic disease as a comparative model for investigating biofilm ecology, antimicrobial tolerance, and novel therapeutic strategies in rabbit odontogenic infections [11,22,60].

Striking parallels can be drawn between rabbit odontogenic abscesses and human dental plaque and osteomyelitis-associated biofilms, particularly with respect to biofilm architecture, microbial interactions, and persistence mechanisms. Human dental plaque represents one of the best-characterized multispecies biofilm ecosystems, consisting of highly organized consortia of aerobic and anaerobic microorganisms embedded within an EPS matrix. These communities exhibit pronounced spatial stratification, metabolic cooperation, and coordinated gene expression, all of which contribute to ecological stability and resilience under environmental stress [20,21,22,59]. Likewise, chronic osteomyelitis is increasingly recognized as a biofilm-mediated infection in which pathogens such as *Staphylococcus aureus* colonize bone surfaces, evade host immune responses, and survive prolonged antimicrobial therapy through EPS-mediated protection, metabolic adaptation, and persister-cell formation [11,45,48,61].

These features closely resemble those observed in rabbit odontogenic abscesses, where polymicrobial communities persist within a dense, poorly vascularized, caseous environment that favors long-term bacterial survival and recurrence. In all three systems, biofilm-associated bacteria exhibit profound physiological adaptations, including altered gene expression, metabolic heterogeneity, phenotypic tolerance, and enhanced resistance to both host immune defenses and antimicrobial therapy, collectively promoting chronic infection and treatment failure [11,22,45,61]. Importantly, the recurrent detection of bacterial genera such as *Staphylococcus*, *Streptococcus*, *Fusobacterium*, and *Pseudomonas* in both human and rabbit biofilm-associated infections further supports the translational relevance of rabbit odontogenic abscesses as a naturally occurring model for studying polymicrobial biofilm ecology, antimicrobial tolerance, and chronic infection [4,7,20,29,60]. Together, these similarities suggest that rabbit odontogenic abscesses reproduce many of the ecological and functional characteristics of human biofilm-associated infections, supporting their value as a comparative model for investigating microbial persistence, antimicrobial resistance, and the development of innovative anti-biofilm therapies within a One Health framework [13,52,53,62,63].

Periodontal disease in dogs and cats represents another clinically relevant model of chronic polymicrobial biofilm-associated infection and offers valuable comparative insights into rabbit odontogenic abscesses. Similarly to human dental plaque, canine and feline periodontal disease develops through the sequential maturation of complex multispecies biofilms on tooth surfaces, accompanied by ecological shifts from predominantly Gram-positive early colonizers to increasingly diverse anaerobic communities that drive chronic inflammation and progressive destruction of periodontal tissues [20,59,64]. Rather than being caused by a single pathogen, periodontal disease results from dysbiosis within the oral microbiome, where microbial interactions, biofilm maturation, and host inflammatory responses collectively determine disease progression [64,65].

In contrast to canine and feline periodontal disease, rabbit odontogenic abscesses are typically initiated by underlying dental abnormalities, including malocclusion, apical tooth elongation, trauma, or other conditions that permit bacterial invasion of the periapical tissues and surrounding bone rather than superficial plaque accumulation alone [2,15]. Nevertheless, once established, both diseases share fundamental pathogenic mechanisms, including polymicrobial biofilm formation, chronic inflammation, microbial persistence, and reduced susceptibility to antimicrobial therapy, highlighting common ecological principles despite marked differences in disease initiation and dental anatomy and physiology, particularly the elodont and hypsodont dentition of rabbits compared with the anelodont and brachyodont dentition of dogs and cats [11,20,22,65].

Experimental rodent models of mandibular osteomyelitis provide an important translational platform for investigating the pathogenesis of chronic bone infections and evaluating novel anti-biofilm therapeutic strategies. Unlike naturally occurring rabbit odontogenic abscesses, rodent models allow precise control over the infecting microorganism, inoculum size, host genetics, and treatment protocols, facilitating mechanistic studies that cannot be readily performed in clinical patients. Most experimental models employ *Staphylococcus aureus* because of its central role in chronic osteomyelitis and biofilm formation, although polymicrobial models are increasingly being developed to better reproduce the complexity of clinical infections [61,66].

Early experimental models established the feasibility of inducing mandibular osteomyelitis through direct intraosseous bacterial inoculation or hematogenous dissemination. Experimental rat models of mandibular osteomyelitis have been established using direct inoculation of *S. aureus* into surgically created mandibular bone defects [67], whereas Hienz et al. later developed a hematogenous model using intravenous *S. aureus* administration combined with local bone injury [68]. These experimental systems have provided valuable insights into bacterial colonization, biofilm establishment, host inflammatory responses, and bone remodeling during chronic infection, while also serving as platforms for evaluating therapeutic approaches [66].

Although these experimental models do not fully reproduce the polymicrobial ecology and characteristic caseous lesions of rabbit odontogenic abscesses, they offer complementary mechanistic insights into biofilm-associated osteomyelitis. An additional anatomical limitation should be considered when extrapolating from mouse or rat mandibular models, as their cheek teeth are brachyodont and anelodont, whereas rabbit cheek teeth are hypsodont and elodont. Together with naturally occurring rabbit disease, rodent models contribute to a translational research continuum spanning experimental pathogenesis, therapeutic development, and comparative One Health investigations of chronic biofilm-associated infections [11,61,63,66].

Unlike experimental animal models, rabbit odontogenic abscesses develop spontaneously under naturally occurring clinical conditions and therefore more closely reproduce the complexity of chronic biofilm-associated infections encountered in veterinary practice. Their polymicrobial nature, prolonged disease course, repeated antimicrobial exposure, and frequent need for surgical intervention make them a valuable translational model for investigating biofilm persistence, antimicrobial tolerance, and therapeutic strategies that may be applicable across both veterinary and human medicine. However, this translational relevance should be interpreted in the context of the distinctive anatomy and physiology of rabbit dentition, which differ substantially from those of other species. This comparative value extends beyond rabbit health by linking naturally occurring veterinary infections with broader questions of biofilm persistence, antimicrobial use, and resistance that are relevant across animal and human medicine, thereby supporting the integration of rabbit odontogenic infections into a One Health framework [62,63].

The increasing popularity of rabbits as companion animals further strengthens the One Health relevance of these infections. Recent studies have demonstrated that pet rabbits can harbor antimicrobial-resistant bacteria under natural clinical conditions, emphasizing their potential role in the maintenance and dissemination of resistance determinants within household environments. Although most available evidence originates from respiratory and other opportunistic infections rather than odontogenic disease, these findings underscore the importance of prudent antimicrobial use, routine susceptibility testing, and integrated surveillance strategies across companion animal medicine. Similar bacterial genera, including *Staphylococcus*, *Pseudomonas*, and *Pasteurella*, are implicated in multiple rabbit infections, suggesting that antimicrobial resistance should be considered at the host level rather than as an isolated feature of a single clinical syndrome [69,70].

From a One Health perspective, antimicrobial resistance surveillance in companion rabbits should extend beyond individual clinical syndromes to encompass integrated monitoring of bacterial populations circulating across animals, humans, and the environment. Expanding coordinated surveillance initiatives, such as EARS-Vet, together with responsible antimicrobial stewardship, routine culture and susceptibility testing, and improved diagnostic approaches, could help limit the emergence and dissemination of antimicrobial resistance in companion animal practice [70,71]. Thus, the One Health relevance of rabbit odontogenic infections lies not only in their comparative value as naturally occurring biofilm-associated diseases but also in the need to integrate antimicrobial stewardship, resistance surveillance, and responsible infection management across veterinary, human, and environmental health contexts. The principal similarities and differences between rabbit-specific and human evidence regarding polymicrobial infection, biofilm biology, antimicrobial tolerance, and resistance are summarized in Table 2, with particular emphasis on areas in which rabbit-specific evidence remains limited and interpretation relies on comparative data.

## 8. Current and Emerging Anti-Biofilm Strategies

Systemic antimicrobial therapy may serve as an adjunctive and supportive component of the management of rabbit odontogenic abscesses but should not be considered a standalone treatment because biofilm formation, extensive tissue necrosis, and poor vascularization markedly limit antimicrobial penetration. Consequently, successful treatment primarily depends on adequate source control through surgical debridement, elimination of the underlying dental lesion, and appropriate postoperative local wound management, with systemic antimicrobial therapy used as an adjunct when indicated. When adjacent bone or soft tissues are involved, adequate source control may also require removal of infected or necrotic tissue, while persistent or recurrent infection should prompt reassessment for a residual infectious focus rather than reliance on prolonged antimicrobial therapy alone. Because rabbits are particularly susceptible to antimicrobial-induced gastrointestinal dysbiosis, antimicrobial selection should prioritize both efficacy and safety while, whenever possible, being guided by bacterial culture and susceptibility testing. The principal systemic antimicrobial agents currently used in rabbits, together with their main advantages, limitations, and clinical considerations, are summarized in Table 3.

Overall, available rabbit clinical evidence indicates that systemic antimicrobial therapy alone should not be regarded as definitive treatment for chronic odontogenic abscesses, because adequate source control generally requires removal of the underlying dental and infected or necrotic tissues, while mature biofilms, caseous exudate, and poor vascularization may further reduce antimicrobial efficacy. Consequently, antimicrobial therapy should be regarded primarily as an adjunct to surgery and local biofilm control rather than as a substitute for definitive surgical management. In addition, prolonged empirical antimicrobial administration should be avoided whenever possible to minimize selective pressure for antimicrobial resistance and support antimicrobial stewardship [3,4,15].

Following thorough surgical debridement, local antimicrobial delivery may be used as an adjunct to postoperative local wound management in selected rabbit odontogenic abscesses. Compared with systemic therapy alone, local antimicrobial delivery achieves substantially higher drug concentrations at the infection site while minimizing systemic exposure and partially overcoming the limited antimicrobial penetration associated with biofilm formation and poor tissue vascularization [15,72].

The two principal local delivery systems described in rabbits are biodegradable collagen sponges impregnated with antimicrobial agents and antibiotic-impregnated polymethylmethacrylate (PMMA) beads [15,72]. Collagen sponges provide temporary local antimicrobial release and are gradually resorbed, eliminating the need for surgical removal, although drug elution is relatively short-lived. In contrast, PMMA beads provide sustained local release of high antimicrobial concentrations over a prolonged period and are particularly useful in chronic infections associated with osteomyelitis or residual contaminated tissues [15,29,72,73]. However, because PMMA is non-biodegradable, the beads may require subsequent removal once antimicrobial elution is complete, as they can persist as foreign material and potentially serve as a nidus for recurrent infection [15,29,72,73]. Overall, current evidence supports the use of local antimicrobial delivery as an adjunct to meticulous surgical debridement rather than as a replacement for definitive surgical management, particularly in chronic biofilm-associated odontogenic abscesses.

Gentamicin-loaded PMMA beads are the most frequently described local antimicrobial delivery system in rabbits because they provide sustained release of high local antibiotic concentrations for several weeks and exhibit activity against common pathogens associated with rabbit odontogenic abscesses, particularly *Staphylococcus* spp. and *Pseudomonas* spp. [2,15,29]. Amikacin has also been used as an alternative aminoglycoside for local delivery because of its broad activity against Gram-negative bacteria, including *Pseudomonas* spp., while minimizing systemic exposure when incorporated into PMMA beads [74]. To maximize therapeutic efficacy, the beads should be placed in direct contact with the infected bone, as antibiotic diffusion into the surrounding tissues is limited to only a few millimeters [73]. Despite these advantages, PMMA is a non-biodegradable carrier and, once antimicrobial elution is complete, the beads may persist as a foreign body and potentially serve as a nidus for recurrent infection. Consequently, surgical removal should be considered if recurrence develops, whereas asymptomatic implants may be left in situ [15,74].

Following surgical debridement, several adjunctive local therapies have been described to support the management of open abscess cavities in rabbits. These include calcium hydroxide, medical-grade honey, hyperosmotic sugar preparations, hydrogel wound dressings (e.g., Intrasite Gel), and bioactive ceramic materials [73,75]. Although these agents differ in composition and mechanism of action, their primary objectives are to promote wound healing, facilitate tissue regeneration, reduce the residual bacterial burden, and create a local environment less favorable for biofilm persistence. However, evidence supporting their efficacy is derived predominantly from case reports, case series, and clinical experience rather than controlled clinical studies, and none should be regarded as a substitute for meticulous surgical debridement and elimination of the primary dental lesion [15,29].

Medical-grade honey has been used as an adjunctive local treatment for open rabbit abscess cavities following adequate surgical debridement. Its therapeutic effects are attributed to multiple mechanisms, including high osmolarity, low pH, hydrogen peroxide production, and the presence of bioactive phytochemicals, which collectively inhibit bacterial growth, disrupt biofilm formation, and promote tissue repair and wound healing [2,72,76,77]. Successful application requires complete drainage of purulent material, meticulous debridement, elimination of necrotic tissue, and correction of the underlying cause, such as extraction of the affected tooth or removal of foreign material [15,76]. Honey is applied directly into the abscess cavity rather than onto the skin surface and may be administered repeatedly during postoperative wound management until healthy granulation tissue develops [76]. Although encouraging clinical outcomes have been reported in rabbits, current evidence remains largely based on case reports and clinical experience, and controlled studies evaluating its efficacy in rabbit odontogenic abscesses are still lacking [15,76]. Beyond its antimicrobial properties, honey has been shown to interfere with biofilm formation and to increase the susceptibility of biofilm-associated bacteria to antimicrobial agents, making it an attractive adjunctive therapy for chronic odontogenic infections.

## 9. Emerging Anti-Biofilm Therapies and Future Perspectives

The therapeutic approaches discussed in this section are supported predominantly by preclinical evidence from in vitro studies and experimental models, with limited early clinical evidence in human medicine for selected strategies. None of these emerging anti-biofilm approaches has yet been specifically evaluated in clinical or experimental studies of rabbit odontogenic abscesses. Enzymatic disruption of the EPS matrix has emerged as one of the most promising anti-biofilm strategies because it targets the structural integrity of the biofilm rather than directly killing bacterial cells. Matrix-degrading enzymes hydrolyze key EPS components, including extracellular DNA (eDNA), polysaccharides, and structural proteins, thereby destabilizing biofilm architecture, facilitating bacterial detachment, and enhancing the penetration and efficacy of both antimicrobial agents and host immune defenses [78,79,80]. Among the most extensively investigated enzymes are deoxyribonuclease I (DNase I), which degrades extracellular DNA, Dispersin B, which hydrolyzes poly-β-(1,6)-N-acetyl-D-glucosamine (PNAG), alginate lyase, which targets alginate-rich biofilms produced by *Pseudomonas aeruginosa*, and various proteases that degrade protein components of the biofilm matrix [78,79,80]. In addition, carbohydrate-degrading enzymes such as mutanase and dextranase have shown promising activity against oral biofilms by degrading extracellular glucans that contribute to biofilm stability, although current evidence is largely limited to in vitro studies [81].

Preclinical and predominantly in vitro studies indicate that enzyme-based approaches may be most effective as adjuncts to conventional antimicrobial strategies rather than as standalone treatments, as disruption of the EPS matrix markedly improves antimicrobial penetration into mature biofilms [78,79,80]. Although enzyme-based strategies have shown encouraging results in experimental models of oral, orthopedic, and chronic wound biofilms, their application in rabbit odontogenic abscesses has not yet been investigated. Consequently, further studies are required to evaluate their safety, stability, and clinical efficacy before they can be considered for routine veterinary use in rabbits [78,79,80].

QS inhibition has emerged as a promising anti-biofilm strategy by disrupting bacterial communication rather than directly targeting bacterial viability. QS regulates multiple processes essential for biofilm development, including EPS production, biofilm maturation, virulence factor expression, motility, stress adaptation, and antimicrobial tolerance [39,40,44]. Consequently, inhibition of QS signaling can attenuate bacterial pathogenicity, impair biofilm formation, and increase the susceptibility of biofilm-associated microorganisms to both antimicrobial agents and host immune defenses [44,78,79,80]. Unlike conventional antibiotics, quorum-sensing inhibitors (QSIs) generally exert little or no direct bactericidal activity, thereby potentially reducing the selective pressure associated with the emergence of antimicrobial resistance [44,78]. A broad range of naturally derived and synthetic QSIs has been investigated, including halogenated furanones, ajoene derived from *Allium sativum*, flavonoids, plant polyphenols, antimicrobial peptides, and synthetic small-molecule inhibitors that interfere with autoinducer synthesis or receptor binding [78,79,80]. Preclinical studies, particularly in vitro investigations, have shown that these compounds can reduce biofilm biomass, suppress the expression of virulence-associated genes, and enhance the activity of conventional antimicrobial agents against clinically important biofilm-forming pathogens, including *Pseudomonas aeruginosa* and *Staphylococcus aureus* [44,78,79,80,81,82]. However, despite encouraging in vitro and experimental findings, QSIs have not yet been evaluated in rabbit odontogenic abscesses. Their future clinical application will require further studies to establish their safety, pharmacological properties, and therapeutic efficacy in naturally occurring rabbit biofilm-associated infections [78,79,80,81,82].

Bacteriophage therapy has re-emerged as a promising strategy for the treatment of chronic biofilm-associated infections, particularly those caused by multidrug-resistant bacteria. Unlike conventional antibiotics, lytic bacteriophages selectively infect susceptible bacterial cells, replicate within them, and induce bacterial lysis while largely preserving the commensal microbiota [78,79,80,83]. In addition to their direct antibacterial activity, many bacteriophages produce polysaccharide depolymerases and other biofilm-degrading enzymes that facilitate disruption of the EPS matrix, thereby improving phage penetration into mature biofilms and enhancing the efficacy of concomitantly administered antimicrobial agents [78,79,80,83].

Preclinical studies and early clinical experience in human medicine have reported encouraging results in the treatment of chronic osteomyelitis, implant-associated infections, and chronic wound infections caused by biofilm-forming pathogens, particularly *Pseudomonas aeruginosa* and *Staphylococcus aureus* [78,79,80,83]. Moreover, combination therapy with bacteriophages and conventional antibiotics frequently exhibits synergistic effects, resulting in enhanced biofilm eradication, reduced bacterial burden, and a lower likelihood of resistance emergence compared with either approach alone [78,79,80,83]. Although these findings are encouraging, bacteriophage therapy has not yet been investigated in rabbit odontogenic abscesses. Consequently, further studies are required to evaluate phage selection, host specificity, delivery strategies, safety, and therapeutic efficacy before this approach can be considered for clinical application in naturally occurring rabbit biofilm-associated infections [78,79,80,83].

Antimicrobial peptides (AMPs) have attracted considerable attention as potential anti-biofilm agents because of their broad-spectrum antimicrobial activity and multiple mechanisms of action. Unlike conventional antibiotics, AMPs primarily disrupt bacterial cell membranes through electrostatic interactions, resulting in rapid bacterial killing while also exhibiting immunomodulatory and anti-inflammatory properties [78,79,80]. In addition to their direct antimicrobial effects, several AMPs interfere with biofilm formation by inhibiting initial bacterial adhesion, disrupting the EPS matrix, and reducing quorum-sensing-mediated signaling, thereby increasing the susceptibility of biofilm-associated bacteria to both antimicrobial agents and host immune defenses [78,79,80]. Preclinical studies, predominantly in vitro, have demonstrated activity against clinically important biofilm-forming pathogens, including *Pseudomonas aeruginosa*, *Staphylococcus aureus*, and *Enterococcus faecalis*, with several peptides also showing synergistic effects when combined with conventional antibiotics [78,79,80]. Despite these promising findings, important challenges remain, including peptide instability, susceptibility to proteolytic degradation, potential cytotoxicity, and the high cost of large-scale production, all of which currently limit clinical application [78,79,80]. Moreover, AMPs have not yet been evaluated in rabbit odontogenic abscesses. Further investigations are therefore required to determine their pharmacological properties, safety, and therapeutic efficacy before they can be considered as adjunctive therapies for chronic biofilm-associated infections in rabbits [78,79,80].

Nanotechnology-based approaches have emerged as promising strategies for overcoming the limitations of conventional antimicrobial therapy in chronic biofilm-associated infections [80,84,85,86]. Nanomaterials can improve antimicrobial delivery, facilitate penetration through the EPS matrix, provide sustained local drug release, and enable targeted delivery of antimicrobial agents directly to biofilm-associated bacteria [84,85,86]. Depending on their composition, nanoparticles may also exhibit intrinsic antimicrobial activity through membrane disruption, generation of reactive oxygen species, interference with bacterial metabolism, and modulation of quorum-sensing pathways involved in biofilm development [84,85,86]. Metallic nanoparticles, including silver, zinc oxide, and copper nanoparticles, as well as polymeric, lipid-based, and bioinspired nanocarriers, have demonstrated significant activity in preclinical studies against clinically important biofilm-forming pathogens such as *Staphylococcus aureus* and *Pseudomonas aeruginosa*, while also enhancing the efficacy of conventional antimicrobial agents when used in combination [84,85,86]. In addition, multifunctional nanomaterials capable of simultaneously delivering antimicrobial compounds and disrupting biofilm architecture represent an emerging strategy for improving treatment efficacy against persistent biofilm-associated infections [84,85,86]. Despite these promising experimental findings, concerns regarding cytotoxicity, long-term biocompatibility, manufacturing scalability, regulatory approval, and potential environmental impacts remain important barriers to clinical translation [85,86]. Furthermore, nanotechnology-based anti-biofilm therapies have not yet been investigated in rabbit odontogenic abscesses. Consequently, further studies are required to establish their safety, pharmacokinetic characteristics, and therapeutic efficacy before clinical application can be considered in naturally occurring rabbit biofilm-associated infections [80,84,85,86].

## 10. Research Gaps and Future Directions

To facilitate translation of current knowledge into clinically relevant advances, future research priorities can be structured according to short-, medium-, and long-term objectives. As a short-term research priority, standardized in vitro and ex vivo biofilm models using clinical rabbit isolates should be developed to address important methodological gaps in rabbit-specific biofilm research. Most current knowledge regarding biofilm formation, antimicrobial susceptibility, and the evaluation of emerging anti-biofilm therapies is derived from human medicine or experimental models involving other bacterial species and host systems. The absence of validated rabbit-specific biofilm models limits the reproducibility of experimental findings, hinders direct comparisons between studies, and complicates the preclinical assessment of novel antimicrobial and biofilm-disruptive strategies. Establishing standardized experimental models that accurately reproduce the polymicrobial composition, EPS architecture, and microenvironment of naturally occurring rabbit odontogenic abscesses would substantially improve translational research and facilitate the development of evidence-based therapeutic approaches [87,88,89].

A complementary short-term priority is the integration of multi-omics approaches to provide a comprehensive characterization of rabbit odontogenic biofilms. Combining metagenomics, transcriptomics, proteomics, metabolomics, and resistome analysis would enable simultaneous investigation of microbial community composition, functional activity, metabolic adaptations, and antimicrobial resistance determinants within naturally occurring infections. Such integrated analyses could identify key molecular pathways associated with biofilm maturation, persistence, and treatment failure while improving our understanding of host–microbe interactions and the ecological complexity of polymicrobial communities. Applying these technologies to rabbit odontogenic abscesses would strengthen translational comparisons with human biofilm-associated diseases, facilitate the identification of novel therapeutic targets, and support the development of more precise diagnostic and antimicrobial stewardship strategies within a One Health framework [90,91,92].

As a medium-term priority, well-designed prospective and longitudinal clinical studies integrating microbiological, molecular, and clinical data should be conducted to improve the management of rabbit odontogenic abscesses. Correlating microbial community composition, biofilm architecture, antimicrobial resistance profiles, and host immune responses with treatment outcomes would facilitate the identification of prognostic biomarkers associated with disease persistence, recurrence, and therapeutic success. Longitudinal studies incorporating standardized sampling protocols before, during, and after treatment could further clarify the temporal dynamics of biofilm-associated communities and antimicrobial resistance under therapeutic pressure. Such integrated clinical investigations would strengthen evidence-based treatment protocols, optimize antimicrobial stewardship, and enhance the translational value of rabbit odontogenic infections as a comparative model for chronic biofilm-associated diseases within a One Health framework [93,94,95].

As a long-term priority, research should focus on the clinical translation and validation of innovative anti-biofilm therapeutic strategies for rabbit odontogenic infections. Beyond conventional antimicrobial therapy, emerging approaches such as antimicrobial photodynamic therapy, antimicrobial peptides, bacteriophage therapy, quorum sensing inhibitors, nanoparticles, and biofilm-disrupting agents warrant systematic evaluation using standardized experimental and clinical models. These strategies should be assessed not only for their antimicrobial efficacy but also for their ability to disrupt mature polymicrobial biofilms, reduce antimicrobial resistance selection, preserve the beneficial oral microbiota, and improve long-term clinical outcomes. Integrating these novel therapeutic modalities with evidence-based antimicrobial stewardship principles may provide more effective and sustainable management of chronic odontogenic infections while further strengthening the translational relevance of the rabbit model for both veterinary and human oral medicine [78,79,80,81,82,83,84,85,86,96,97,98].

## 11. Limitations of Rabbit-Specific Evidence

Despite the growing clinical and microbiological interest in rabbit odontogenic infections, important limitations remain in the available rabbit-specific evidence. Direct characterization of biofilm architecture, quorum-sensing activity, persister-cell formation, efflux-pump involvement, and other mechanisms of biofilm-associated antimicrobial tolerance in naturally occurring rabbit odontogenic abscesses remains limited. Consequently, several mechanistic concepts discussed in this review are based partly on evidence from human dental infections, experimental osteomyelitis models, and broader biofilm research and should therefore be interpreted as biologically plausible rather than definitively demonstrated in rabbits. In addition, much of the available rabbit literature consists of retrospective clinical studies, case series, or culture-based microbiological investigations, with relatively few prospective studies incorporating standardized sampling, advanced molecular characterization, and long-term imaging-confirmed outcomes. These limitations emphasize the need for rabbit-specific experimental and longitudinal clinical studies to validate the proposed mechanisms and strengthen evidence-based therapeutic recommendations.

## 12. Conclusions

Rabbit odontogenic infections are complex, chronic polymicrobial diseases in which the characteristic caseous, poorly vascularized lesion environment and biofilm-associated bacterial persistence can substantially reduce antimicrobial efficacy and contribute to treatment failure and recurrence. Effective management therefore requires a multimodal approach centered on accurate diagnosis, elimination of the underlying dental source, appropriate surgical debridement and local management, together with culture- and susceptibility-guided antimicrobial therapy and responsible antimicrobial stewardship. The biological similarities between these infections and human endodontic and other chronic biofilm-associated diseases also support their comparative relevance within a One Health framework.

Important knowledge gaps nevertheless remain, particularly because direct rabbit-specific evidence for biofilm architecture, quorum-sensing activity, persister-cell formation, and several proposed antimicrobial resistance mechanisms is still limited. Future research should prioritize standardized rabbit-specific experimental models, prospective longitudinal clinical studies, and integrated molecular, imaging, and multi-omics approaches to better define microbial community dynamics, resistance determinants, host–microbe interactions, and treatment responses. Validation of emerging anti-biofilm strategies in rabbits will also be essential to translate mechanistic insights into safe, evidence-based therapies that improve long-term clinical outcomes and rabbit welfare while advancing understanding of chronic biofilm-associated infections across animal and human health.

## Figures and Tables

**Table 1:** Bacterial species associated with rabbit odontogenic abscesses and their microbiological, biofilm-related, and antimicrobial resistance characteristics [4,7,16,17,29].

Bacterial Species	Gram Stain	Oxygen Requirement	Role in Odontogenic Abscesses	Biofilm-Related Evidence	Common Resistance Concerns
*Pasteurella multocida*	Gram−	Facultative anaerobe	Common rabbit pathogen; reported in rabbit infections	Biofilm-forming capacity reported; rabbit odontogenic evidence limited	Generally susceptible to conventional antimicrobials; resistance may occur
*Staphylococcus aureus*	Gram+	Facultative anaerobe	Reported in rabbit abscesses and other infections	Well-established biofilm former; rabbit odontogenic evidence limited	Multidrug resistance reported
*Pseudomonas aeruginosa*	Gram−	Aerobic	Reported in rabbit abscesses and other infections	Well-established biofilm former; rabbit odontogenic evidence limited	Intrinsic and acquired multidrug resistance; efflux-mediated resistance recognized
*Stenotrophomonas maltophilia*	Gram−	Aerobic	Opportunistic pathogen reported in rabbits	Biofilm-forming capacity recognized; rabbit odontogenic evidence limited	Intrinsic multidrug resistance
*Fusobacterium nucleatum*	Gram−	Obligate anaerobe	Anaerobic component of polymicrobial odontogenic abscesses	Established biofilm-associated species; rabbit-specific biofilm evidence limited	Variable antimicrobial susceptibility
*Prevotella* spp.	Gram−	Obligate anaerobe	Anaerobic component of polymicrobial odontogenic abscesses	Biofilm-associated; rabbit-specific biofilm evidence limited	Variable antimicrobial susceptibility
*Actinomyces* spp.	Gram+	Facultative/anaerobic	Component of chronic polymicrobial odontogenic abscesses	Biofilm-associated; rabbit-specific evidence limited	Variable antimicrobial susceptibility
*Streptococcus* spp.	Gram+	Facultative anaerobe	Component of polymicrobial odontogenic infections	Well-established biofilm-associated genus; rabbit-specific evidence limited	Variable antimicrobial susceptibility
*Escherichia coli*	Gram−	Facultative anaerobe	Occasional opportunist isolate in rabbits	Biofilm-forming capacity reported; rabbit odontogenic evidence limited	Multidrug resistance reported
*Proteus* spp.	Gram−	Facultative anaerobe	Opportunistic isolate reported in rabbits	Biofilm-forming capacity reported; rabbit odontogenic evidence limited	Multidrug resistance reported

**Table 2:** Comparative evidence for major features of odontogenic biofilm-associated infections in rabbits and humans.

Feature	Evidence in Rabbits	Evidence in Humans	Comparative Interpretation
Polymicrobial infection	Odontogenic abscesses contain diverse aerobic and anaerobic bacterial communities [4,7,15,29].	Endodontic infections are well-established polymicrobial diseases characterized by complex microbial communities [20,57,58].	Direct evidence supports polymicrobial infection in both species.
Anaerobic component	Anaerobic taxa, including *Fusobacterium*, *Prevotella*, and *Actinomyces*, have been identified in rabbit odontogenic infections [15,29].	Obligate anaerobes, including *Porphyromonas*, *Prevotella*, *Fusobacterium*, and *Treponema*, are major components of mature human endodontic microbiota [20,57,58,59].	Anaerobic bacteria are important in both species, although microbial composition differs between hosts.
Biofilm formation and organization	Bacterial species with recognized biofilm-forming capacity have been identified in rabbit infections, but direct characterization of biofilm formation and mature biofilm architecture in rabbit odontogenic abscesses remains limited [4,7].	Structured multispecies dental biofilms and their ecological organization are extensively characterized [20,21,22,59].	Rabbit-specific structural evidence is more limited, and detailed mechanistic interpretation therefore relies partly on comparative evidence.
Lesion microenvironment	Dense caseous exudate, fibrous encapsulation, and poor vascularization characterize rabbit abscesses [15,16].	Mature dental biofilms exhibit spatial and physicochemical heterogeneity, including oxygen and nutrient gradients [20,22].	Rabbit lesions provide biologically plausible conditions for similar gradients, but direct rabbit-specific demonstration remains limited.
Quorum sensing	A specific role for QS in rabbit odontogenic abscesses has not been directly established [4,7].	QS-mediated regulation of biofilm development, microbial interactions, and virulence is well characterized in relevant bacterial biofilm systems [39,40,41,42,43,44].	Its proposed role in rabbit odontogenic disease remains primarily extrapolative.
Persister cells and phenotypic tolerance	Persister cells have not been directly demonstrated in rabbit odontogenic abscesses	Phenotypic tolerance and physiologically heterogeneous bacterial subpopulations are well characterized in chronic odontogenic and biofilm-associated infections [45,46].	Their involvement in rabbits should currently be regarded as a biologically plausible hypothesis rather than an experimentally demonstrated mechanism.
Antimicrobial resistance and biofilm-associated tolerance	AMR has been documented in bacteria isolated from pet rabbits, while direct mechanistic evidence specifically linking biofilm-associated resistance mechanisms to rabbit odontogenic disease remains limited [4,5].	Genetic and phenotypic mechanisms underlying AMR and biofilm-associated tolerance are extensively characterized in biofilm research [11,31,32,33,34,35,36,37,38].	

**Table 3:** Systemic antimicrobial agents used in rabbit odontogenic abscesses: advantages, limitations, and clinical considerations [3,4,15,29].

Antimicrobial	Typical Route	Advantages	Limitations	Clinical Considerations
Enrofloxacin	Oral or injectable	Licensed for systemic use in rabbits in some jurisdictions; good activity against several Gram-negative pathogens.	Limited evidence of efficacy against chronic rabbit odontogenic abscesses; antimicrobial resistance has been reported	Should preferably be selected based on culture and susceptibility testing rather than empirical use.
Penicillin (injectable formulations)	Parenteral	Clinical efficacy has been reported in rabbits with odontogenic abscesses; less disruption of intestinal microbiota than oral administration.	Should not be used as a substitute for adequate surgical debridement; prolonged treatment may be required	Frequently considered one of the preferred systemic options when administered parenterally.
Amoxicillin (injectable)	Parenteral	Parenteral administration is less likely to disrupt intestinal microbiota than oral administration	Limited evidence specifically for rabbit odontogenic abscesses.	Should not replace appropriate surgical management.
Cefalexin (injectable)	Parenteral	Parenteral administration is less likely to disrupt intestinal microbiota than oral administration	Limited rabbit-specific evidence.	Injectable administration is preferred over oral use.
Marbofloxacin	Oral	Good tissue penetration and oral bioavailability; convenient administration.	Limited rabbit-specific evidence in chronic odontogenic abscesses	Best used when supported by susceptibility testing.
Trimethoprim–sulfadiazine	Oral	Generally well tolerated; broad antimicrobial spectrum	Activity may be reduced by purulent exudate and debris; low susceptibility has been reported among isolates from rabbit odontogenic abscesses	Use should preferably be guided by culture and susceptibility testing
Doxycycline	Oral	Useful against selected susceptible organisms	Limited evidence for odontogenic abscesses;	Consider only when indicated by culture and susceptibility testing.
Clindamycin/Lincomycin	Oral	Commonly used in other species for osteomyelitis	Contraindicated because of severe gastrointestinal dysbiosis and enterotoxemia risk	Direct placement into abscess cavities is not recommended because accidental ingestion may be fatal.

## Data Availability

No new data were created or analyzed in this study.
